# Diagnosis of myxedema coma complicated by renal failure: a case report

**DOI:** 10.1002/ccr3.850

**Published:** 2017-02-21

**Authors:** Akiteru Takamura, Ryusho Sangen, Yoshiki Furumura, Daisuke Usuda, Yuji Kasamaki, Tsugiyasu Kanda

**Affiliations:** ^1^ Department of General Medicine CenterKanazawa Medical University Hospital1‐1 UchinadaKahoku‐gunIshikawaJapan; ^2^ Department of Medical EducationKanazawa Medical University1‐1 UchinadaKahoku‐gunIshikawaJapan; ^3^ Department of Family MedicineMie University Graduate School of Medicine2‐174 Edo‐bashiTsuMieJapan; ^4^ Department of General MedicineHimi Municipal HospitalKanazawa Medical University31‐9 Saiwai‐choHimiToyamaJapan

**Keywords:** Case report, diagnosis, emergency, myxedema coma, renal failure

## Abstract

Myxedema coma, caused by severe lack of thyroid hormone, is characterized by deterioration of mental status, hypothermia, hypotension, hyponatremia, and hypoventilation. We describe an 84‐year‐old woman who presented with renal failure and new onset severe hypothyroidism leading to challenges in the recognition of myxedema coma.

## Introduction

Myxedema coma is typically a sequela of chronic severe thyroid hormone deficiency and is characterized by deterioration of mental status, hypothermia, hypotension, hyponatremia, and hypoventilation. It is one of the most urgent and lethal endocrine conditions. Criteria and scoring system for the diagnosis of myxedema coma, based on specific clinical features and laboratory data, have been proposed, but no consensus has been reached. However, early recognition and commencement of treatment based on clinical suspicion is recommended, rather than waiting for laboratory results 1, 2. A history of thyroidectomy, radioiodine therapy, or sometimes just hypothyroidism may be important clues of myxedema coma. Few studies to date, however, have assessed the association between myxedema coma and renal failure.

Although decompensated hypothyroidism is a rare endocrine emergency, but differentiation from other, similar conditions are important for critically ill patients presenting with systemic illness 3, 4. Myxedema coma is one of the most severe potential consequences of profound, long‐standing hypothyroidism. Alternatively, myxedema coma may be triggered by an acute event such as infection (including sepsis), heart attack, stroke, injury, cold exposure, or the administration of drugs 5. Importantly myxedema coma particularly affects the elderly age group 6. However, in patients with pre‐existing chronic disease such as renal failure, and no prior history of thyroid disease, the recognition of myxedema coma may be particularly challenging due to the overlap in the clinical and laboratory features of severe hypothyroidism and renal failure.

## Case Report

An 84‐year‐old Japanese woman with renal failure of unknown cause for a few years, and who was not taking any medications and supplements, presented to a primary care clinic with generalized pain of a few days’ duration, without abnormal mentation. She was initially diagnosed with deterioration in renal failure due to dehydration and was referred to and treated with amlodipine 2.5 mg, furosemide 40 mg, and calcium polystyrene sulfate 10 g without dialysis at the Department of Nephrology in our hospital. Over the following 6 weeks, her activities of daily living deteriorated and finally she became comatose and was taken to the Emergency Department.

On initial physical examination, the patient was pale, with a body temperature of 36.6°C. Her initial blood pressure was 133/72 mmHg, her heart rate was 56 beats/min, and her oxygen saturation was 92%. She demonstrated very slow mentation (Glasgow Coma Scale: 8, Japan Coma Scale III‐100) and was somnolent, but able to be aroused. Her mucous membranes were dry, there was no thyroid goiter, her heart sounds were distant, her lungs were clear, and her abdomen was not distended. Her lower extremity pulses were weak and pretibial nonpitting edema was present. However, her renal function was not worse than that on her previous admission.

**Figure 1:**
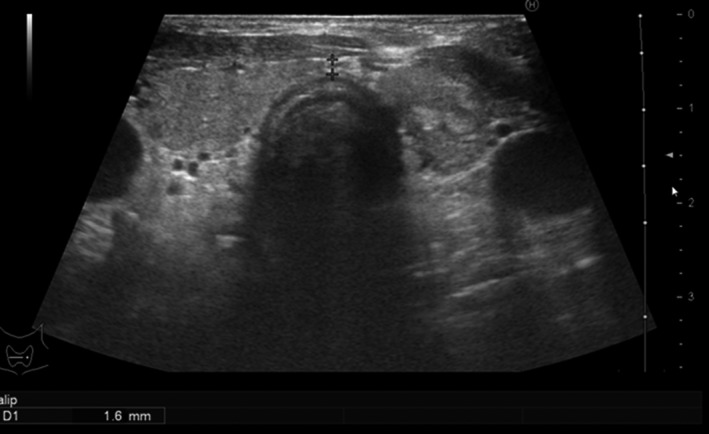
Ultrasound showing that this patient had a normal‐sized thyroid with small nodules on admission.

**Figure 2:**
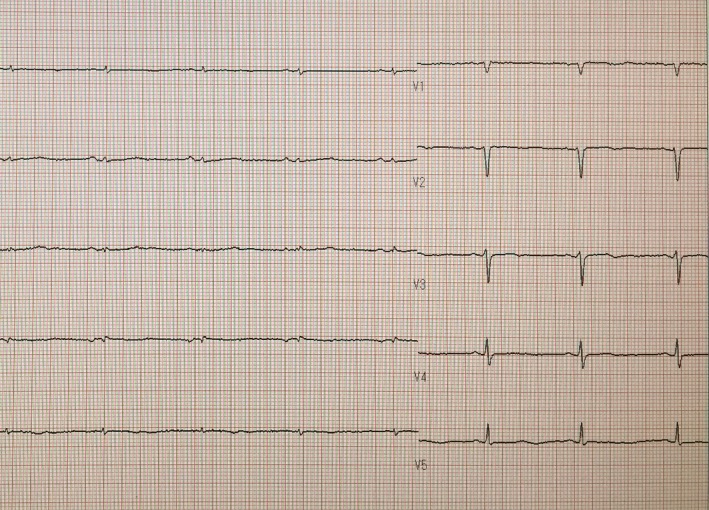
Electrocardiogram showing low voltage on admission.

**Figure 3:**
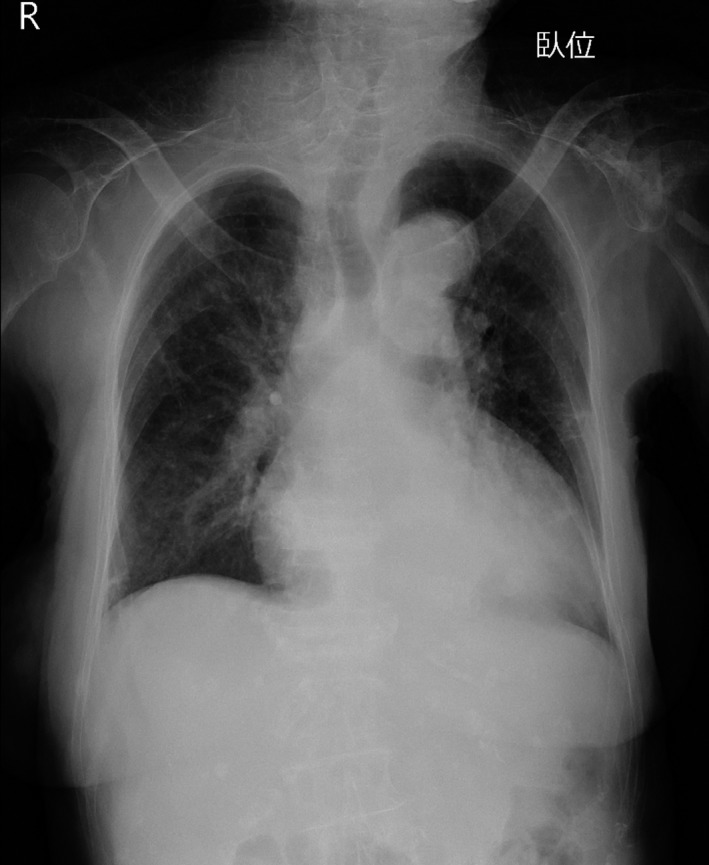
Chest X‐ray showing cardiomegaly and pleural effusion on admission.

Laboratory investigations on admission showed hypothyroidism, mild anemia, elevated serum creatine kinase concentration, and impaired renal function (Table 1). Liver function and electrolytes were within normal ranges; that is, she was not hyponatremic. Blood gas analysis showed slight hypocapnia, but her oxygen level was within normal range. Blood ammonia was also within normal range, and thyroid antibodies were detected (Table 2). Ultrasonography showed normal echoic findings with normal size and small nodules of thyroid (Fig. 1). An X‐ray (Fig. 2) and a computed tomography scan (Fig. 3) showed slight cardiomegaly and pleural effusion, but no abnormalities in the lung fields. An electrocardiogram revealed all waves were low voltage (Fig. 3).

**Table 1:** Laboratory data on admission

Variables	Normal reference range	Results
Hemoglobin (g/dL)	12–16	8.6
Red blood cells (/*μ*L)	3.8–4.8 × 10^7^	2.41
MCV (fl)	84–99	105
MCHC (%)	30.2–35.1	34.2
Platelet count (/*μ*L)	15–45 × 10^4^	11.8 × 10^4^
White cell count (/*μ*L)	4.0–9.0 × 10^3^	3.5 × 10^3^
Neutrophils (/*μ*L)	1.8–7.5 × 10^3^	2.8 × 10^3^
AST (IU/L)	10–34	44
ALT (IU/L)	5–46	38
CK (IU/L)	40–150	839
TSP (g/dL)	6.6–8.1	6.2
BUN (mg/dL)	8–20	70.1
CRE (mg/dL)	0.46–0.79	4.47
Sodium (mEq/L)	138–145	142
Potassium (mEq/L)	3.6–4.8	5.1
Chlorine (mEq/L)	101–108	106

MCV, mean cell volume; MCHC, mean corpuscular hemoglobin concentration; AST, aspartate aminotransferase; ALT, alanine aminotransferase; CK, creatine phosphokinase; TSP, total serum protein; BUN, blood urea nitrogen; CRE, creatinine.

**Table 2:** Laboratory data on admission

Variables	Normal reference range	Results
pH	7.5 to 7.45	7.43
PaCO_2_ (mmHg)	74 to 104	34.1
PaO_2_ (mmHg)	38 to 44	89.3
HCO_3_ (mEq/L)	22 to 26	22.3
Base excess (mEq/L)	−2 to +2	−1.3
Anion gap	12	11.4
TSH (*μ*IU/mL)	0.35 to 4.94	200<
Free T_3_ (pg/mL)	1.71 to 3.71	0.59
Free T_4_ (ng/mL)	0.70 to 1.48	0.05
TgAb (U/mL)	0.3>	14.5
TPOAb (U/mL)	0.3>	8.6
NH_3_ (*μ*g/dL)	30 to 86	53
BNP (pg/mL)	20>	80.4
Cortisol (*μ*g/dL)	4.0 to 23.9	18.8

TgAb, anti‐thyroglobulin antibody; TPOAb, anti‐thyroid peroxidase antibody; BNP, brain natriuretic peptide.

These findings suggested myxedema coma. The patient was started on treatment with oral levothyroxine sodium hydrate (100 *μ*g/day) and intravenous hydrocortisone sodium succinate (100 mg every 8 h), in case of coexisting adrenal insufficiency. The patient was administered sufficient oxygen to treat her relative hypoxemia. Dialysis was not implemented. There was no evidence of hypotension, hypothermia, or hyponatremia. Her mental condition improved dramatically within a few days along, with the normalization of thyroid hormone. Treatment with glucocorticoid and oxygen was gradually tapered off, and the patient was discharged 3 weeks later with only oral medication. Following treatment, her serum creatine kinase concentration decreased from 839 to 56 mg/dL, as did her creatinine level from 4.47 to 4.26 mg/dL.

## Discussion

Thyroid hormone plays a key role in regulating the body's metabolic processes. Myxedema coma is an infrequent life‐threatening complication of hypothyroidism 7. Myxedema coma may be part of the natural progression of hypothyroidism or may be triggered in patients with chronic hypothyroidism by conditions such as drug overdose, myocardial infarction, cerebral infarction, injury, or cold exposure with previously existing chronic hypothyroidism 7, 8, 9. It is thought to most commonly affect elderly women. Hypothyroidism is present in about 6% of the population to varying degrees, but myxedema coma is seen in only 0.1% of this subgroup of patients 7. However, a previous study reported that 39% of patients with myxedema had no history of hypothyroidism 10. It still retains a high mortality rate of almost 40–50% despite aggressive treatment 11, 12.

In clinical practice, myxedema coma is often diagnosed based on symptoms and physical examination, without waiting for laboratory results. Among the most significant features of myxedema coma are its cardiovascular complications 7, characterized by low voltage complexes on electrocardiogram 11, 12. Hypothyroid patients are often hypotensive due to decreased cardiac output, and patients with severe hypothyroidism may experience both decreased heart rate and myocardial contractility 7. Although the patient described here did not have low blood pressure, hypothermia, or hyponatremia, her respiratory function was slightly impaired and her consciousness was affected. Hypoxemia has been described hypoxemia in 80% and hypercapnia in 54% of myxedema crisis patients 13.

Our patient had normal blood oxygen but was hypocapnic, a finding thought to result from relative hyperventilation to compensate for the metabolic acidosis caused by her chronic renal failure. Therefore, we regarded this patient as having relative hypoxemia. The length of time the patient had been in renal failure and the extent to which her renal condition had affected her consciousness and respiratory function could not be determined. However, her hypoxemia and hypocapnia might have been masked for some time.

There have been few clinical reports to date of myxedema complicated with renal failure. Metabolic disorders of the thyroid, particularly hypothyroidism, are frequent in patients with chronic kidney disease and end‐stage renal disease. In addition, the kidney and the thyroid play key roles in the metabolism, degradation, and excretion of thyroid hormone and its metabolites 12. Primary hypothyroidism has been associated with a consistent elevation in serum creatinine levels 14, 15. Several studies have suggested that serum TSH be examined when screening patients with renal failure 14, 16, 17.

Deterioration in renal function was found to correlate with the occurrence of hypothyroidism 16, 18. Several hypotheses have been proposed to explain the mechanism by which serum creatinine levels are increased in hypothyroid patients. According to these hypotheses, hypercreatinemia is secondary to the renal dysfunction associated with hemodynamic changes that occur in primary hypothyroidism. Altered thyroid function induces a reduction in cardiac output, while increasing peripheral resistance, leading to systemic and renal vasoconstriction. This effect reduces renal blood flow and, therefore, glomerular filtration rate 14, 16, 17, 19, 20. We could not determine whether hypothyroidism preceded or followed renal failure. Consequently, we could not determine whether hypothyroidism preceded or followed renal impairment. Although this may be challenging to distinguish in practice, careful attention to detail and awareness of the possibility of hypothyroidism in patients with renal failure will improve outcomes. In this case, appropriate management of hypothyroidism resulted in improvement in renal function 8.

## Conclusion

In summary, we described a rare case of myxedema coma with atypical features associated with renal failure. The patient presented with mild respiratory failure and a reduced level of consciousness without hypotension, hypothermia, or hyponatremia. Myxedema is rarely experienced in our practice. However, many reports have described patients on dialysis have co‐existing hypothyroidism. Therefore, myxedema coma must be considered in the differential diagnosis. Treatment should be started when a patient presents with coma and chronic kidney disease with or without dialysis, even in the absence of hypotension, hypothermia, hyponatremia, and hypoxemia.

## Conflict of Interest

None declared.

## Declarations

Ethics approval and consent to participate. This report was approved by the Ethics Committee of Kanazawa Medical University Himi Municipal Hospital.

## Consent for Publication

Written informed consent was obtained from the patient for publication of this case report and the accompanying images. A record of the consent form is available for review by the editor‐in‐chief of this journal.

## Authorship

AT, RS, YF, DU, YK, and TK: contributed equally to the care of this patient. AT: wrote the manuscript. All the authors read and approved the final manuscript.
